# Manipulating the diseased oral microbiome: the power of probiotics and prebiotics

**DOI:** 10.1080/20002297.2024.2307416

**Published:** 2024-01-31

**Authors:** X. Yu, D.A. Devine, J.J. Vernon

**Affiliations:** Division of Oral Biology, School of Dentistry, University of Leeds, Leeds, UK

**Keywords:** Metabolomics, oral health, caries, periodontal disease, probiotics, prebiotics

## Abstract

Dental caries and periodontal disease are amongst the most prevalent global disorders. Their aetiology is rooted in microbial activity within the oral cavity, through the generation of detrimental metabolites and the instigation of potentially adverse host immune responses. Due to the increasing threat of antimicrobial resistance, alternative approaches to readdress the balance are necessary. Advances in sequencing technologies have established relationships between disease and oral dysbiosis, and commercial enterprises seek to identify probiotic and prebiotic formulations to tackle preventable oral disorders through colonisation with, or promotion of, beneficial microbes. It is the metabolic characteristics and immunomodulatory capabilities of resident species which underlie health status. Research emphasis on the metabolic environment of the oral cavity has elucidated relationships between commensal and pathogenic organisms, for example, the sequential metabolism of fermentable carbohydrates deemed central to acid production in cariogenicity. Therefore, a focus on the preservation of an ecological homeostasis in the oral environment may be the most appropriate approach to health conservation. In this review we discuss an ecological approach to the maintenance of a healthy oral environment and debate the potential use of probiotic and prebiotic supplementation, specifically targeted at sustaining oral niches to preserve the delicately balanced microbiome.

## Key messages

- The balance of oral homeostasis requires delicate adjustments to prevent and counteract disease.
- The metabolic activities of the complete microbiome, not only key pathogens or commensals, are important to the maintenance of health.
- Metabolomics techniques can be valuable in identifying environmental niches deficient in disease that can act as targets for probiotic and prebiotic treatments.

## Introduction

In the age of extensive antimicrobial resistance, effective replacements for traditional antibiotic therapies are extremely valuable to clinicians from all disciplines, including dentistry. Driven by a strong commercial push, probiotics and prebiotics are promising alternatives for maintaining the delicate balance between host and microbiome, necessary to health.

Most research conducted in the field of probiotics and prebiotics has focused on the gastrointestinal system and its impact on health, as well as local and systemic diseases [1–7.] However, the influence of pro- and prebiotics on the activity of the oral microbiome is understudied. There is a paucity of data outlining the precise impact and mechanisms of potentially beneficial oral microbes. Since the oral microbiome is not only crucial to oral health, but is also linked with many systemic diseases, such as diabetes mellitus [8,9], rheumatoid arthritis [10–13], cardiovascular disease [14,15] and Alzheimer’s disease [16–18], the need for adjunctive therapies to maintain microbial homeostasis is clear.

**Figure 1.**
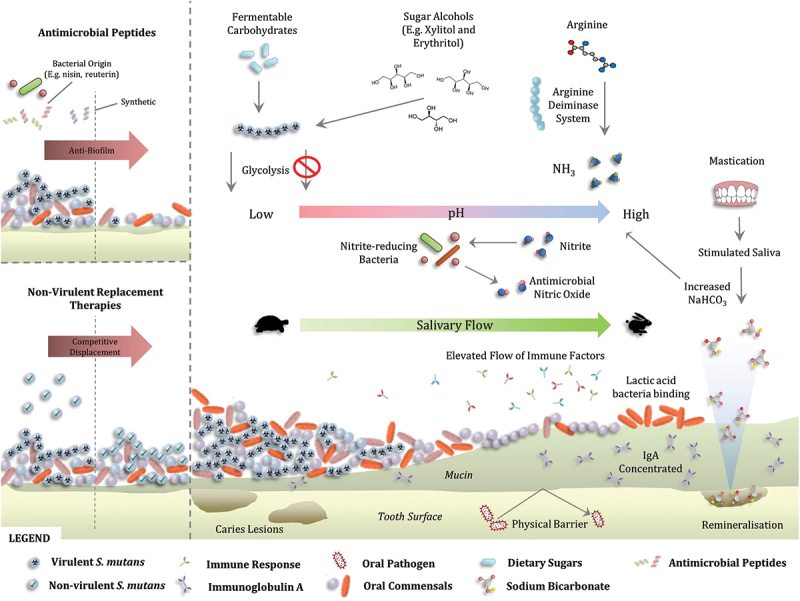
Approaches for the preservation of a healthy oral ecology.

The relationship between host and resident oral microorganisms is one of synergy, with dysbiosis often responsible for disease onset. In 1994 Marsh proposed the ‘ecological plaque hypothesis’, underlining the significance of bacterial interactions, both with other microorganisms and the host environment [19]. This highlighted the importance of collaborative metabolic activity within the microbiome and its impact on health. Modern sequencing and ‘omics’ technologies have facilitated the identification of species associated with health and disease. However, the importance of each organism is the contribution it makes to a succession of metabolic events essential to nutritional, environmental and immunological balance (Figure 1). Modulation of both the microbial composition and collective metabolic activity is central to an ecological approach towards preserving health [20–22]. Hence, the development of pro- and prebiotic solutions can be informed through assessment of metabolic capabilities, facilitating generation of therapies capable of inducing delicate adjustments to the oral microbial equilibrium. Here we discuss oral ecology in health and disease, with a focus on the metabolic alterations of pro- and prebiotic therapies for caries and periodontitis.

*Main panel: Arginine is metabolised into ammonia by bacteria exhibiting the arginine deiminase system. The alkaline products neutralise detrimental acid production from dietary fermentable carbohydrates. Sugar alcohols such as xylitol and erythritol competitively inhibit the glycolysis reaction of fermentable carbohydrates by caries-associated pathogens such as S. mutans, minimising the generation of acidic products. Increased mastication can stimulate salivary flow which has multiple benefits: improved removal of loosely bound bacteria, increased flow of immune elements, increased release of sodium bicarbonate capable of contributing to remineralisation of the enamel and increasing pH, and a quicker reinstatement of a neutral oral pH. Up-regulation of mucin production or a reduced breakdown of mucin-related glycan by inhibiting bacteria capable of doing so can result in an increased physical barrier and a concentration of IgA, hindering invading pathogens. Probiotic lactic acid bacteria are capable of preferential binding on oral surfaces reducing the prevalence of pathogens. Dietary nitrates, metabolised into nitrites in the gut and concentrated in the serum can be reduced by nitrite-reducing bacteria in the mouth, generating nitric oxide, which can confer antimicrobial properties onto the oral communities. However, these can also stimulate an immune response from gingival cells potentially causing periodontal deterioration. Top left panel: Antimicrobial peptides, of bacterial, host and synthetic origin can target communities and convey anti-biofilm effects. Specifically targeted antimicrobial peptides (STAMPs) can impart a more precise targeted effect directly on pathogens, exploiting bacterial signals including pheromones. Bottom left panel: Replacement therapies use isogenic bacteria with attenuated virulence to competitively inhibit and displace virulent strains from oral biofilms.*

## Probiotic and prebiotic definitions

Since inception of the term ‘probiotics’ [23], its definition has been revised to reflect scientific advances. The nomenclature has broadened to include the symbiotic stimulation of other organisms through secreted compounds [24], before being expanded to the current concept of live organisms conferring a host health benefit [25,26]. Prebiotics were first defined as ‘non-digestible food ingredients providing benefits through selective metabolism in the gut’ [2], e.g. inulin, lactulose, fructo-oligosaccharides and galacto-oligosaccharides [27]. An expanded definition that omits the limitation to the gut and includes elements influencing microbiome composition and metabolic state ensures a more relevant, holistic classification [28,29].

Definitions have advanced and delineated further, with many nascent phrases cited in the literature: Live Biotherapeutic Products, Next Generation Probiotics, Probioceuticals, Paraprobiotics, Synbiotics and Symbiotics [26]. Since focusing on the metabolic balance of the oral environment is of primary importance, for the purpose of this review we will refer to any beneficial approach using live bacteria or growth enhancing compounds as ‘probiotic’ or ‘prebiotic’, respectively.

## Probiotics for the oral microbiome

Clinical trials have been investigating the influence of probiotics from an oral disease perspective for over twenty years [30–43]. However, no consensus has been reached as to the true efficacy of this approach, with recent multiple systematic reviews and meta-analyses concurring on beneficial trends but identifying the requirement for further studies [44–50].

The majority of the research has concentrated on the genus *Lactobacillus*, since these bacteria have demonstrated efficacy in the gut against systemic diseases [51]. *Lactobacillus reuteri*, *Lactobacillus rhamnosus* and *Lactobacillus acidophilus* are amongst the species exhibiting beneficial effects on both caries [31,37,52,53] and periodontal disease [40,42,54–56]. This genus has a strong potential for maintaining the balance of oral microbiome observed in health. Nevertheless, other genera have demonstrated beneficial effects which may indicate further targets for health preservation [32,33,57–59].

## Prebiotics for the oral microbiome

Prebiotics can help to promote the growth of beneficial, commensal bacteria and maintain a healthy balance of microorganisms in the mouth, through several metabolic pathways. However, as with probiotics, many common prebiotics are targeted at gut microbiota [60–62]. Subsequently, the attention for oral prebiotics has mainly focused on distinct compounds, often with local applications.

Slomka and colleagues used high-throughput Phenotype MicroArrays to screen a series of compounds for use as potential oral prebiotics [63]. They identified two compounds capable of selectively boosting commensal organisms in the mouth, N-acetyl-D-mannosamine and beta-methyl-D-galactoside. In two-species competitive biofilm cultures, both compounds stimulated increased growth and a population shift in favour of the commensal organisms. Interestingly, beta-methyl-D-galactoside has been demonstrated to interfere with the co-aggregation of oral pathogens, whilst N-acetyl-D-mannosamine is part of the sialic acid metabolism pathway [64]. Orthologs of N-acetyl-D-mannosamine have been reported in several oral streptococci, including *S. gordonii*, *S. mitis* and *S. oralis* [63].

Nitrates also have potential as prebiotics capable of influencing the oral environment. A range of oral commensal bacteria, including *Veillonella* and *Actinomyces* spp., play an essential role in the nitrate-nitrite-nitric oxide metabolic pathway [65,66]. These nitrate-reducing bacteria utilize dietary/salivary nitrate and produce nitrite, assisting further generation of nitric oxide, important in maintaining oral and systemic health [67]. An *in vitro* study assessing the short-term influence of nitrate on complex oral biofilms indicated that within nine hours of supplementation there was a significant increase in ammonium and an associated shift in pH [68]. Observed through 16S rRNA gene sequencing, this alteration resulted in significant increases in nitrate-reducing bacterial genera, *Rothia* and *Neisseria*, and an associated decrease in periodontitis, halitosis, and caries-associated pathogens.

It is important to note that the research on dental prebiotics and their impact on metabolism is still in the early stages, and more research is necessary. However, the current evidence suggests that dental prebiotics may have the potential to promote metabolic health and prevent the development of certain metabolic disorders [63,68–70].

## Probiotics and prebiotics in dental caries

Dental caries is the most prevalent noncommunicable disease globally [71]. Cariogenic bacteria in dental plaque, such as *Streptococcus mutans*, metabolise fermentable dietary carbohydrates producing acid, which decreases pH and causes enamel demineralization over time [72,73]. Targeting these processes is key to the efficacy of pro- and prebiotic approaches to combating caries.

## Probiotics for the prevention of dental caries

Oral probiotics research has often focused heavily on the reduction of cariogenic organisms, specifically *S. mutans*. Since the metabolic cascade and virulence properties of *S. mutans* are well-characterised, a diverse range of approaches have been taken. These include competitive inhibition with non-pathogenic commensals, the introduction of species capable of alternative carbohydrate fermentation, or the addition of mutant knock-out strains with an absence of virulence genes.

### *In vitro* studies

*In vitro* studies can identify prospective candidate probiotic strains and are useful in outlining potentially contributory mechanisms. Reports from one South Korean group indicated that *Lactobacillus lactis* reduced biofilm formation *in vitro*, which correlated with decreased *S. mutans* counts and importantly, reduced levels of glucosyltransferases [57,74]. A reduction in these crucial caries virulence factors can result in attenuated glucan production, a key component of a cariogenic biofilm [75]. Furthermore, there is a body of evidence linking *Bifidobacterium* with a beneficial reduction in *S. mutans* populations [76–79]. *In vitro* reports of this genus’s ability to form complexes with *Fusobacterium* on hydroxyapatite surfaces denote a potential favourable influence on biofilm formation [80] This affinity for coaggregation with a key bridging organism may be beneficial to colonisation and retention of the probiotic, in turn, competitively inhibiting *S. mutans*.

### *In vivo* studies

To understand the holistic impact on oral ecology we must look to *in vivo* trials. Probiotic instillations have demonstrated efficacy by priming the immune system to deal with pathogenic threats [41,81,82]. One recent study of 50 participants demonstrated elevated IgA concentrations in a group administered a probiotic lozenge of *Lactobacillus salivarius* subs. *salicinius*, *Lactobacillus paracasei* and *Lactobacillus plantarum* [81]. This correlated with substantially reduced oral pathogen populations, including *S. mutans*, indicating the importance of immune stimulation in anti-caries, probiotic effects. Other lactobacilli also appear to have an inhibitory effect on oral pathogens, with reductions in both caries lesions and *S. mutans* populations observed in children consuming probiotic milk products [31,37]. These benefits are generally associated with reduced plaque formation, potentially due to stimulation of the immune system. Similarly, other probiotic organisms including *Bifidobacterium* and *Bacillus* strains have also been strongly associated with an indirect modulation of inflammatory response [41,58,82–85].

The use of commensal and non-pathogenic *Streptococcus* species has also been trialled as probiotic agents [34,86,87]. Hedayati-Hajikand *et al*. observed significant reductions of early caries indications in 2–3-year-old children consuming *Streptococcus rattus*, *Streptococcus oralis* and *Streptococcus uberis* enriched chewing tablets [34]. The authors proposed that this combination eradicated *S. mutans* through both competitive inhibition and the production of hydrogen peroxide. This corroborated earlier animal model experiments which indicated that a lactate dehydrogenase (LDH) deficient *S. rattus* strain was capable of displacing *S. mutans* on oral surfaces [88]. In the absence of the LDH enzyme, pyruvate can be metabolised down alternative pathways (pyruvate-formate lyase or pyruvate dehydrogenase), subsequently forming pH neutral substances, such as ethanol, tempering cariogenicity [89]. Reduced cariogenicity was also observed in a rat model harbouring a LDH-deficient *S. mutans* strain [90]. These studies demonstrate the potential for exploiting oral bacterial mutants, deficient in key virulence factors, as potential replacement therapies.

### Bacterial replacement therapy

Bacterial replacement therapeutics is an area of research eliciting substantial attention [88,91–95]. The concept is based on employing oral bacterial variants, deficient in virulence factors, as a method to substitute pathogens for harmless isogenic bacteria. This approach has value in causing less disruption and potentially less collateral damage to the oral microbiome. Hillman has suggested that a strain selected for replacement therapy must abide by four tenets: 1 – a vastly attenuated pathogenesis potential, 2 – a significant capability to outcompete the pathogenic strain for the environmental niche, 3 – ability to displace pre-existing, colonising pathogens, 4 – confirmation of safety from any detrimental effects, whether local or systemic [93]. Much of the research in this field has been directed at caries prevention and the use of *S. mutans* variants [89,92,95–97]. Hillman identified a LDH-deficient *S. mutans* strain with a greatly attenuated ability to produce acid, demonstrating reductions of caries lesions in rats by 90% [92]. Crucially, the authors also indicated the persistent colonisation and retention of the non-pathogenic strain. More recently, a Chinese group built upon the use of LDH-deficient strains by further knocking out a negative regulator gene, *gcrR*, for glucan-binding lectin expression, deemed important for adhesion and biofilm formation [94,95]. Here they demonstrated elevated adhesion in the replacement strain compared to wild-type *S. mutans*, significantly reducing caries *in vivo* [94]. One study investigated the potential of a non-virulent *S. mutans* strain with heightened bacteriocin production, capable of killing almost all other *S. mutans* strains [98]. Promisingly, the research revealed that individuals with an initial high level of cariogenic *S. mutans* were free from the pathogen up to two and a half years post-replacement therapy. This study demonstrated the strong appeal and potential of replacement treatments and their superior longitudinal effects compared to standard probiotic therapies. However, the risks of horizontal gene transfer and spontaneous mutation must be considered carefully with this approach. Furthermore, the introduction into the oral milieu of pre-existing antimicrobial resistance determinants harboured by probiotic strains must be considered for the future of antimicrobial efficacy. Highlighting the importance of this point, a recent study assessing the antimicrobial resistance profiles of strains contained within commercially available probiotic lozenges identified genes potentially conveying resistance to more than 30 antibiotics [99].

Interesting advances in the replacement therapy approach have been proposed, with the potential use of bacterial auxotrophs examined. By designing genetic variants with an auxotrophic requirement for D-amino acids, a replacement organism could be eliminated if necessary, alleviating some of the risk [91]. Since D-amino acids are not naturally available, the use of these strains would require dietary supplementation, but ultimately offers the safety net to eliminate the therapeutic strain via cessation of D-amino acid administration. Nonetheless, much like the blockbuster film Jurassic Park (1993) [100], where a lysine contingency was established to control genetically procreated dinosaurs, unforeseen circumstances can occur, although in this case the threat might be oral acidification as opposed to being eaten alive.

Playful digressions aside, replacement therapies present a promising alternative to classical probiotics, offering the incentive of minimal further dysbiosis of the oral ecology. Nonetheless, extensive clinical trialling and safety evaluations of the potential reactivation of virulence through gene transfer or mutation are necessary.

### Long-term probiotic retention

Long-term retention and colonisation of the oral cavity by beneficial bacteria is crucial to disease prevention. Nonetheless, caution must be taken with this approach and any potential risks of local and systemic disorders must be considered. Many studies have attempted to introduce beneficial species, particularly in early childhood, but often the microbiome relapses soon after probiotic intake ceases [31,37,52]. Further longitudinal studies are essential to identify this crucial characteristic in prospective probiotic strains. In order to better understand retention and long-term efficacy, several parameters should be adhered to during longitudinal studies. Implementing frequent and consistent sample collections over an extended period, particularly after cessation of probiotic intake, would allow for population variations to be monitored. Conducting comprehensive microbiome analyses at each time point would improve identification of low abundance presence, whilst monitoring of additional demographics such as antibiotic usage, diet and comorbidities would provide further insight. Furthermore, as longitudinal research often suffers from participant drop out, powering the study size with attrition rates in mind would enable stronger outcomes [101].

## Prebiotics for the prevention of dental caries

### Arginine

The amino acid arginine has versatile effects on human wellbeing, including the enhancement of wound healing [102], immune responses [103,104], cardiovascular function [105], and oral health [106]. In the oral cavity, arginine exists in saliva as free acid or as peptide constituents and has been included in some oral hygiene products [107,108]. Many oral bacteria, especially the genus *Streptococcus*, including *S. gordonii*, *S. sanguinis*, *S. parasanguinis*, *S. cristatus*, *S. mitis* and *S. oralis* [109,110], are able to utilize arginine. It is catabolized by these bacteria via the arginine deiminase (ADS) or agmatine deiminase system (AgDS), both of which pathways generate ammonia and ATP [111]. However, AgDS activity is relatively lower than that of ADS [112], so it is postulated that AgDS-produced ammonia does not contribute sufficiently to oral alkalization [113]. Conversely, the ammonia released via ADS can create an alkaline environment and thus neutralize acids produced by bacterial fermentation of dietary carbohydrates, inhibiting enamel demineralization [111]. Several clinical trials performed by Nascimento *et al*. have found an inverse correlation between ADS activity and caries [114–116]. Furthermore, the exposure to arginine also increased ADS activity in the caries-active cohort.

Moreover, arginine can positively affect the balance of oral microbial populations. One study of 45 individuals demonstrated that toothpaste supplemented with 1.5% arginine promoted a favourable compositional shift in supragingival plaque of a caries-active group towards that of a caries-free group [115]. Zheng *et al*. further confirmed this finding and observed that arginine enriched *S. sanguinis* and markedly decreased *S. mutans* levels in saliva and dental plaque [117]. In another (small) study with nine healthy volunteers, toothpaste containing 8% arginine significantly increased the abundance of lactate fermenter, *Veillonella* in saliva [118]. Similarly, Kuriki *et al*. reported that using mouthwash supplemented with 8% arginine significantly increased ammonium ion concentration in saliva and decreased the genera *Atopobium* and *Catonella* that can act as opportunistic pathogens [119]. Notably, the cohort sizes in these studies were relatively small, with two trials only having nine and ten participants. Additionally, many of these studies were funded by industry, which may raise concerns of research bias [120].

### Fluoride

In the last decades, fluoride has been used as an anti-caries agent in several formats, of which fluoride toothpaste is the most widespread globally and has a substantial caries prevention effect [121–123]. The underlying mechanisms have been well discussed and illustrated in the previous studies, including the inhibition of glycolysis to reduce acid production in caries-related *Streptococcus* strains [124,125] and the promotion of remineralization and prevention of demineralization of dental enamel [126,127]. Recent studies have also found that it influences biofilm composition [128–130]. López-López and Mira revealed that health-associated *Streptococcus salivarius* and *Rothia mucilaginosa* were significantly increased when saliva-derived biofilms were exposed to fluoride [130]. In a long-term cohort study, the use of fluoride induced an ecological shift, with increased activity of health-associated streptococci, as demonstrated by metagenomic and metatranscriptomic analysis [131].

Supplementation with additional prebiotics has been demonstrated to enhance the anti-caries efficacy of fluoride. In three-species *in vitro* biofilms, the combination of arginine and fluoride suppressed the growth of acidogenic *S. mutans*, whereas it boosted the growth of *S. sanguinis* (an arginine utiliser and alkaline producer) [132,133]. Furthermore, a number of clinical studies have demonstrated that the use of dentifrice containing both fluoride and arginine resulted in ammonia production, a higher dental plaque pH [134,135] and greater reductions of caries lesions than fluoride alone [106,136,137]. Carda-Diéguez *et al*. found that patients who brushed with fluoride and arginine dentifrice had lower levels of caries- and periodontitis-related bacterial species after six months, compared to the baseline [131]. This synergism could be valuable for caries prevention.

## Probiotics and prebiotics in periodontitis

Periodontitis is an inflammatory gum disease which damages gingival tissue, resulting in compromised attachment and tooth loss [138]. The pathogenesis of periodontal disease is complex and multifactorial. The main causative factor is the accumulation of plaque, leading to the outgrowth of a consortium of periodontal pathogens and dysbiosis of oral biofilms [139]. Although maintenance of good oral health is foremost in the prevention of periodontal diseases, the use of compounds and organisms to counteract the microbial dysbiosis has exhibited adjunctive effects [35,56,140].

## Probiotic compounds for the prevention of periodontitis

Central to periodontal disease is the contribution of de-regulated host immunity, and subsequent selection of an inflammophilic microbial community [141], leading to the degradation of tissue, the periodontal ligament and bone [142]. A range of commensal oral bacteria can attenuate immune responses [143,144] and some probiotic approaches have aimed to harness these properties in periodontitis treatment. *In vitro* work by Ma *et al*., reported decreased cytokine production in human epithelial cell lines exposed to *L. reuteri* [82] and Cosseau *et al*. found that the probiotic strain *S. salivarius* K12 down-regulated cytokine secretion from respiratory and oral keratinocyte cell lines and primary cells [145]. The mechanism of such immune modulation postulated in *in vitro* studies is that these probiotic strains inhibit the translocation of NF-κB subunit into the host cell nucleus and thus prevent the production of proinflammatory cytokines [82,145]. Twetman *et al*. investigated the effects of gum supplemented with *L. reuteri*, observing decreases in both TNFα and CXCL8 pro-inflammatory elements in the gingival crevicular fluid of individuals with moderate gingivitis [41]. Several other clinical trials support these findings, reporting changes in the balance of pro- and anti-inflammatory cytokines in diseased patients undergoing probiotic therapy [58,140,146].

Effective as adjunctive therapy to scaling and root polishing (SRP), the use of *L. reuteri* has displayed both bactericidal and bacteriostatic properties against periodontal pathogens [84]. The production of reuterin, an antimicrobial peptide and metabolite of glycerol, has been implicated as central to the adjunctive effect in periodontitis. Reuterin has been demonstrated to provoke an oxidative stress effect on the oral environment by modification of protein thiol groups [84]. Several studies report reductions in prevalence of disease-associated bacteria, such as *S. mutans* and *Porphyromonas gingivalis* associated with *L. reuteri*. [44,53,55] These may be attributed to the antimicrobial effect of reuterin, but also co-aggregation with pathogenic bacteria as observed by Saha *et al* [53]. or competitive adhesion as demonstrated in lactobacilli contributing to Yakult’s gastrointestinal probiotics [147,148].

### Long-term probiotic retention

As with caries, the concept of longitudinal retention is essential to periodontal probiotics. Vivekananda *et al*. and Tekce *et al*. investigated the use of a *L. reuteri* lozenge as an adjunctive therapy alongside professional plaque removal for the treatment of periodontal disease. They reported superior clinical characteristics in the probiotic groups [40,42] and also reduced re-colonisation rates of obligate anaerobes in the treatment arm one year after probiotic administration. However, they could only demonstrate retention of *L. reuteri* in 11/20 patients by day 90 and in no patients by day 180 [40]. Although low *L. reuteri* retention rates were reported by Tekce *et al*., the detection methodologies relied on agar-based culture and metabolic detection of reuterin [40]. Quantification using this method may be subject to variability and may not provide precise quantitative data, impacting the reliability of the reported retention rates. Furthermore, the sensitivity of reuterin detection methods may vary, and low sensitivity could lead to an underestimation of the actual reuterin production. Incorporating more advanced molecular techniques, such as quantitative polymerase chain reaction (qPCR) or metagenomic approaches, can enhance the specificity and sensitivity of probiotic detection. By employing a combination of detection methods and parameters, including molecular, culture, and viability assessments, a more comprehensive understanding of probiotic retention can be achieved. Another recent trial by Morales *et al*. demonstrated decreased clinical attachment loss, bleeding on probing and probing depth after a one year follow up of those consuming *L. rhamnosus* SP1 (a.k.a. GG) [54]. Whilst these follow-up studies are promising, many more longitudinal studies are necessary to elucidate the true retention rate and therefore the long-term value of these species.

## Prebiotic compounds for the prevention of periodontitis

Prebiotic effects are an understudied discipline outside of the gut environment. However, certain compounds may have value to the oral microbiome when delivered locally, or even systemically processed through the digestive system. Arginine inhibits co-aggregation between periodontal pathogens, including interactions between *P. gingivalis* and *Prevotella oris*/*Prevotella intermedia*/*Treponema denticola* [149,150]. Studies of *P. gingivalis* and *P. intermedia*/*T. denticola* demonstrated that gingipains and/or gingipain-adhesion complexes on the surface of *P. gingivalis* encoded by gingipain genes were involved in the coaggregation and suggested that arginine may work as a potential inhibitor of gingipains which inhibited co-aggregation reactions. Such bacterial co-aggregation is an important factor for adherence, colonization and biofilm formation [151].

Other dietary elements have implications in the dental environment, including nitrite levels. Metabolised from nitrate, nitrite is concentrated in the saliva from the blood plasma and has been putatively implicated in associations with periodontal disease [152]. This has been hypothesized as due to an increased secretory response to the inflamed oral cavity, as a host defence mechanism, whereby the reduction of nitrite to nitric oxide by denitrifying bacteria in the mouth can generate an antimicrobial effect on periodontal pathogens [153,154]. Balance is crucial once again, since denitrification by nitrite-reducing bacteria generates nitric oxide which has antimicrobial abilities [155]. However, it is also involved as a signalling molecule for host inflammatory response in gingival cells, contributing to the tissue destruction associated with periodontitis [152]. These interactions have been further outlined by Wang *et al*. who observed elevated expression of an inducible-nitric oxide synthase gene from host cell interactions with bacterial products of nitrite metabolism [156]. Attaining a homeostatic balance of these compounds may provide an additional angle for the treatment of periodontitis and a return to a healthy microbiome.

## Metabolomics for probiotic and prebiotic discovery

Metabolomics techniques enable a greater understanding of the intricacies of the complex interactions between the host environment and the cohabiting consortia of oral microbes. At present, mass spectrometry and nuclear magnetic resonance are the main techniques in metabolomics and have been employed in the exploration of differential metabolites found in the oral microbiome between health and disease. In periodontal diseases, clinical studies revealed alterations in some metabolites between periodontally healthy and diseased individuals [157–160]. Wei *et al*. observed metabolites related to biosynthesis of amino acid and aminocyl-tRNA, such as isoleucine, hydrocinnamic acid, serotonin, 4-hydroxycinnamic acid and serine, markedly increased in patients with periodontal diseases. Similarly, altered metabolites were identified in children with and without caries, which involved pathways including carbohydrate metabolism [161–163]. By identifying the metabolic pathways associated with health and the deficiencies relating to disease, specific metabolites can be highlighted.

### Metabolomic effects of probiotics in caries

#### Understanding healthy oral metabolism

To understand probiotic effects in disease, we must first identify the desired balance of the metabolic environment. In health, supragingival plaque is nourished with salivary glycoproteins including amino acids, peptides, and mucin. These are processed into sugars and proteins through the action of both host and bacterial glycosidases and proteases. One important environmental condition, pH, is maintained through several well-balanced mechanisms including acid production via sugar catabolism by *Streptococcus*, *Lactobacillus* and *Actinomyces* spp., counteracted by ammonia generation through amino acid metabolism. Furthermore, a constant salivary flow helps sustain the neutrality of the environment [164]. A healthy equilibrium is upheld with a cyclical demineralisation and remineralisation of tooth surfaces, where enamel is protected from acid damage, preventing caries. At homeostasis, dietary sugars metabolised into acids contribute to demineralisation, but calcium and phosphate ions distributed by the saliva, coupled with bacterial alkaline production and the action of lactate converting bacteria, such as *Veillonella*, *Actinomyces* and *Lactobacillus* spp. promote remineralisation [165].

Through analysis of the oral metabolome and proteome, research has begun to elucidate many of the detailed elements of the intra-bacterial and bacterial-host interactions relating to oral diseases. Cyclic in nature, bacterial and host interactions generate metabolites altering the environment and the capabilities of bacterial cells to survive and interact with each other [166].

#### Oral metabolism in disease

Recent studies of the metabolomes of patients with early childhood caries have identified significant differences amongst salivary metabolites [161,167]. Heimisdottir *et al.*’s larger cohort revealed catechin, epicatechin and fucose as strongly correlated with caries, whilst Li *et al*’s 2023 study reported 32 differing metabolites, including those involved in galactose metabolism in *S. mutans*. The latter study pinpointed a complex of four compounds as potentially useful diagnostic biomarkers, 2-benzylmalate, epinephrine, 2-formaminobenzoylacetate and 3-Indoleacrylic acid.

A study from Wen and colleagues compared the metabolic profiles of *S. mutans* and *L. casei* in both mono- and dual-species cultures [168]. UV-HPLC testing of the spent media indicated four-fold reductions in succinic acid in the presence of *L. casei*, suggesting a positive impact on carbohydrate metabolism and a potential valuable mechanism for anti-caries probiotic strains. A recent *in vitro* study investigated the use of *Lactiplantibacillus plantarum* to counteract *S. mutans* growth [169]. In dual-species cultures of *S. mutans* with *Candida albicans*, the inclusion of *L. plantarum* supernatant reduced *S. mutans* biomass. This was associated with reduced carbohydrate metabolism, and high intracellular sucrose levels indicated an accumulation, but not utilisation of sucrose [169]. Furthermore, the authors reported increased concentrations of the sugar alcohols, xylitol, and sorbitol, which may act as less acidogenic substrates.

There is minimal research utilising metabolomics methodologies to investigate the use of probiotics as anti-caries agents. However, one such study by Belda-ferre *et al*. used proteomic analyses to report reductions in LDH and ADS activity in individuals with caries compared to a healthy cohort [170]. Increasing LDH is commonly associated with decreased pH, so these conflicting findings highlight the difficulties in fully understanding the complexities of how probiotic instillations may impact the overall environment. Here, the authors reported greater numbers of sugar transporters in the caries individuals, implying higher levels of sugar intake as a putative explanation. Further complicating the issue, the aforementioned study also identified greater proportions of exopolysaccharide in healthy plaque, which is associated with increased biofilm formation [171]. Further work is essential to elucidate key mechanisms and targets in the search for anti-caries probiotics.

#### Metabolomic effects of prebiotics in caries

The origin of caries lesions has a well-defined cascade of metabolic processes, resulting in acid production from sugar fermentation. Therefore, the introduction of alternative food sources, such as non-fermentable sugars, is a common approach for oral prebiotics. From a therapeutic angle, the use of sugar alcohols such as xylitol has been the subject of some controversy, with systematic reviews and meta-analyses reporting contradictory findings [172–175]. Metabolomics may help us to understand the mechanisms *in vivo*, highlighting the specific impact on oral pathologies.

Takahashi *et al*. determined no effect of 10% xylitol on the plaque metabolome or acid production in a group of Japanese adult volunteers, suggesting that it does not directly inhibit acid production, and may only have an influence through competition as a non-fermentable sugar [176]. Nonetheless, the same study reported a dose-dependent effect of fluoride, reducing lactate production from glucose by up to 46%. Subsequently, there was an increase in 3-phosphoglycerate and a decrease in phosphoenolpyruvate. These changes in 3-phosphoglycerate and phosphoenolpyruvate production indicate an inhibition of enolase activity in the Embden – Meyerhof – Parnas (EMP) pathway, as phosphoenolpyruvate is a product of phosphoglycerate catabolism during glycolysis. Since this process has been identified in cariogenic bacteria such as *S. mutans* [177], the mechanism of acid reduction in the presence of fluoride may be due to interference with the EMP pathway in these pathogens.

A recent study of 83 individuals using either arginine, fluoride or control toothpastes reported increases in supragingival plaque metabolites associated with the maintenance of healthy, non-cariogenic pH. These included glucosamine-6-phosphate, agmatine and phenethylamine in the arginine group. Increases in agmatine could well be linked to the ADS found in commensal streptococci, capable of generating ammonia and neutralising acidic environments [113].

#### Metabolomic effects of probiotics in periodontitis

To identify relevant probiotic strains, we must first consider the metabolic environment of periodontal disease and target the detrimental pathways. Metabolomic studies of periodontitis patients have highlighted increases in components relevant to oxidative stress pathways, lipase, glycosidase and protease activity, and an uptake of saccharides as essential sources of energy for oral bacteria [178,179]. Many of these would contribute to a favourable environment for oral pathogens associated with periodontal disease, such as *P. gingivalis* [180]. Although there is minimal available metabolomics data for the use of probiotics against periodontal disease, by identifying organisms capable of balancing out the biochemical environment, the impact of periodontal diseases may be reduced.

Periodontitis-metabolic biomarkers in saliva, GCF or dental plaque have been investigated for years, but there is no consensus in specific metabolites identified as the disease signature across the studies [157,159,181–183]. Nonetheless, targets might include short chain fatty acids, as butyrate has been demonstrated to impact epithelial cell junctions, amongst other influences [184], protease inhibition to tackle the detrimental proteolytic activity [185] and oxidative stress pathways to counteract the damage caused [186].

Pro- and prebiotics in the oral cavity drive beneficial changes, including inhibiting the virulence gene expression of pathogenic bacteria [187–191], promoting ammonia generation [118] or reducing pro-inflammatory production in host cells [41,82,145,192].

#### Metabolomic effects of prebiotics in periodontitis

There is a paucity of studies assessing the metabolomic impact of oral prebiotics on periodontitis. Comprehensive clinical trials are essential to elucidate the biochemical differences instigated with prebiotic treatments. Potential suitable targets for prebiotics, may come from the signatures identified in periodontitis patients, such as high levels of acetate [193], short-chain fatty acids [194], 2-pyrrolidineacetic acid, butyrylputrescine, dimethylarginine, as well as polyamines and their derivatives e.g. cadaverine [182,195,196]. By introducing prebiotic compounds to promote species with alternative metabolic pathways, or interfere with those associated with diseased states, a potential strategy may be developed to restore periodontal health.

## The future of probiotic and prebiotic research

Throughout the course of this review, we have highlighted the necessity for further research in this field, particularly towards gaining a detailed interpretation of the metabolic environment, through metabolomics and proteomics studies. There is a particular need for well-designed longitudinal studies to allow a better understanding of the dynamic changes in the oral microbiome response to probiotic interventions, over time. Future research focus would benefit from the development of novel probiotic formulations with enhanced stability, targeted delivery mechanisms, and improved viability for optimal oral microbiome modulation. A focus on strain-specific differences in probiotics and their influence on the overall system would enable better precision interventions [197]. Furthermore, there is value in exploring the potential for personalized probiotics and prebiotics tailored to individual oral microbiomes, whilst considering factors such as genetics, lifestyle, and dietary habits. Another research direction is to focus attention to investigating the synergistic effects of combining probiotics with prebiotics (synbiotics) to enhance therapeutic outcomes. By gaining a greater understanding of the underlying mechanisms of probiotic and prebiotic effects, robust links could be established for the amelioration of oral disease and systemic disorders.

## Conclusion

Optimal approaches to the preservation of a balanced healthy oral microbiome may involve complementing many of the well-established dental approaches, such as plaque removal and dietary awareness, with adjunctive therapies. Supplementation with potentially beneficial compounds or colonisation of bacterial species capable of contributing to the healthy metabolic state is one such option. However, treatment of prevalent oral diseases, including caries and periodontitis requires careful ecological manipulation, since each disorder reflects a different set of environmental and metabolic dysbioses, all of which are intrinsically linked. With many ecological properties and effects still unknown and a plethora of systemic diseases demonstrating correlations with the oral environment, achieving a balance of therapies so as not to cause alternate problems remains crucial. The key to this may lie in a greater understanding of a holistic metabolic environment and the modulation of host immune response.
